# Grain orientation and shape evolution of ferroelectric ceramic thick films simulated by phase-field method

**DOI:** 10.1038/s41598-024-67051-4

**Published:** 2024-07-16

**Authors:** Yongmei Zhang, Qingshu Li, Qidong Yue, Ping Wang, Zhenyu Liu

**Affiliations:** 1 College of Information Science and Engineering, Shanxi Agricultural University, Jinzhong, 030801 PR China; 2 College of Agricultural Engineering, Shanxi Agricultural University, Jinzhong, 030801 PR China

**Keywords:** Phase field simulation, Anisotropy, Grain growth, Grain boundary energy, Ferroelectric ceramics, Materials science, Mathematics and computing

## Abstract

The orientation and shape of ceramics grains was always neglected, resulting in a lot of information during sintering has not been excavated. In this study, a modified phase-field model in order to express the anisotropy of grain boundary energy is developed. The effects of the anisotropy of grain boundary energy on the grain orientation and shape evolution are investigated in detail. The ferroelectric ceramic thick films are prepared by tape casting. The comparison of experiment and simulation results shows that the anisotropy of grain boundary energy results in uneven grain orientation and bimodal grain size distribution. The quantitative analysis of grain microstructures helps to establish a relationship with the degree of anisotropy of grain boundary energy. Our findings provide a new way to judge the degree of anisotropy by calculating the relevant parameters in the SEM images of ceramics materials.

## Introduction

Ferroelectric ceramics have been widely used in electronic communication equipment, and the world market for ferroelectric devices is expect to grow up every year^1,2^. In order to improve the stability of products, researchers have been working on the microstructure control of ferroelectric ceramics for a long time^3,4^. Since the grain size has an important effect on the domain structure of ferroelectric ceramics^5,6^, the previous studies focused on the regulation of electrical properties by grain size^7–11^. In fact, according to the theory of ceramic sintering^12^, when the raw materials is fixed of ferroelectric ceramics, the control of grain size is easy to achieve by changing the process parameters. Unfortunately, it is found that the properties of ferroelectric ceramics vary greatly even if the same raw materials were used and the grain size was similar^2,13–19^. The unpredictability of electrical properties can be attributed to the side effects introduced by the uneven distribution of doped ions^4,20–22^. With the successful preparation and rapid development of textured ferroelectric ceramics^9,23–29^, people have realized the importance of grain orientation on the piezoelectric response. So, as a part of microstructure, the grain orientation of ferroelectric ceramics should not be neglected. The study of grain orientation in ferroelectric ceramics may have important scientific significance on optimizing electrical performance.

It is well known that the alignment of ferroelectric domain within polycrystalline ceramics is closely related to the grain orientation^2,9,18,27,30,31^. In general, the orientation of grains during sintering process is randomly distributed. However, due to the asymmetric crystal structure of ferroelectric ceramics materials, the grain boundary energy is obvious anisotropic, which causes the rapid growth of individual grains during the sintering process^32–34^. The bimodal grain size distribution will be formed due to the occurrence of abnormal grain growth, and then the orientation of large grains dominates^35^. In other words, the microstructure of ferroelectric ceramics shows certain texture morphology. Depressingly, these phenomena are difficult to evaluate and investigate experimentally, despite the rapid development of methods for characterizing grain orientation. Modeling and simulations are considered as a promising method to study grain growth process^36–38^. Among these methods, phase-field model, which does not require a continuous tracking interface^39^, is believed to be the efficient method for simulating texture evolution of ceramics^9,29^. Previous numerical studies mostly focus on the grain growth kinetics and change of texture degree during grain coarsening. The grain orientation and shape evolution are lack of insightful investigations.

To experimentally observe grain growth, 3D samples are usually investigated by examining their 2D cross sections. Many efforts have been still devoted to the analytical extraction of 3D microstructural information from cross-sectional observations. So, it is necessary to perform 2D simulations. In this paper, the 2D phase-field method is employed to investigate the grain orientation and shape evolution of ferroelectric ceramic thick films. At present, the grain orientation and shape data could be obtained from the 2D plane, *e.g.* XRD patterns, EBSD, SEM or TEM images. The selection of thick films was mainly to eliminate the influence of thickness on grain growth as much as possible, so as to facilitate the comparison between simulation and experiment results. In addition, ferroelectric ceramic thick films have developed rapidly due to their excellent performance^3,10,14,15,23^. The effect of anisotropy of grain boundary energy on grain microstructure was analyzed in detail.

## Simulation method

### Model description

The phase-field model proposed by Fan and Chen^39^ was often used to simulate the grain growth process. The misorientation and inclination angles were introduced to express anisotropy of grain boundary or interface energy by Moelans et al.^40^. In our previous work^35^, we developed a modified phase-field model to simulate the grain growth process of bulk ceramics. As the transition region between two grains with different orientations (*η*_i_ and *η*_j_), the energy *σ* of the grain boundary was expressed as the sum of the crystal plane energies of the two grains (*γ*_i_ + *γ*_j_). The model of grain boundary energy anisotropy become obviously simplified, thereby reducing the complexity of the solution process and shortening the running time of the program. However, this model cannot quantitatively describe the orientation and shape changes of the target grains, because the growth of grains in ceramics is controlled by 3D space rather than 2D space.

**Figure 1:**
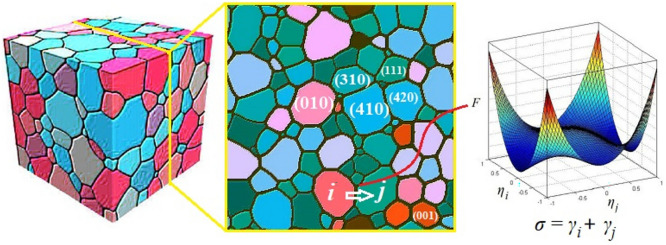
Schematic drawing of a grain boundary formed by two grains with different crystal plane energies.

As shown in Fig. 1, the 2D thick film can be understood as a slice cut from the ceramics bulk. The grain orientations in ferroelectric ceramic thick film can be described by a set of non-conserved, long-range order (LRO) parameters $\eta_{1} (r,t)$, $\eta_{2} (r,t)$, …$\eta_{q} (r,t)$ and *q* as the number of possible grain orientations. The time-dependent Ginzburg–Landau equation for the LRO can be expressed as^39^:

1 $$\frac{\text{d}{\eta }_{i}(r,t)}{\text{d}t}=-{L}_{i}\frac{\updelta F}{\updelta {\eta }_{i}(r,t)}$$

where *L*_*i*_ is the mobility of the grain-boundary migration. The system free energy *F* as a function of LRO parameters can be expressed as follows^39^:

2 $$F={F}_{0}+\int \left[\sum_{i=1}^{q}\left[-\frac{{\eta }_{i}^{2}}{2}+\frac{{\eta }_{i}^{4}}{4}\right]+\sum_{i=1}^{q}\sum_{j>1}^{q}{\eta }_{i}^{2}{\eta }_{j}^{2}+\sum_{i=1}^{q}\frac{{k}_{i}}{2}{\left(\nabla {\eta }_{i}(r)\right)}^{2}\right]{\text{d}}^{3}r$$

**Figure 2:**
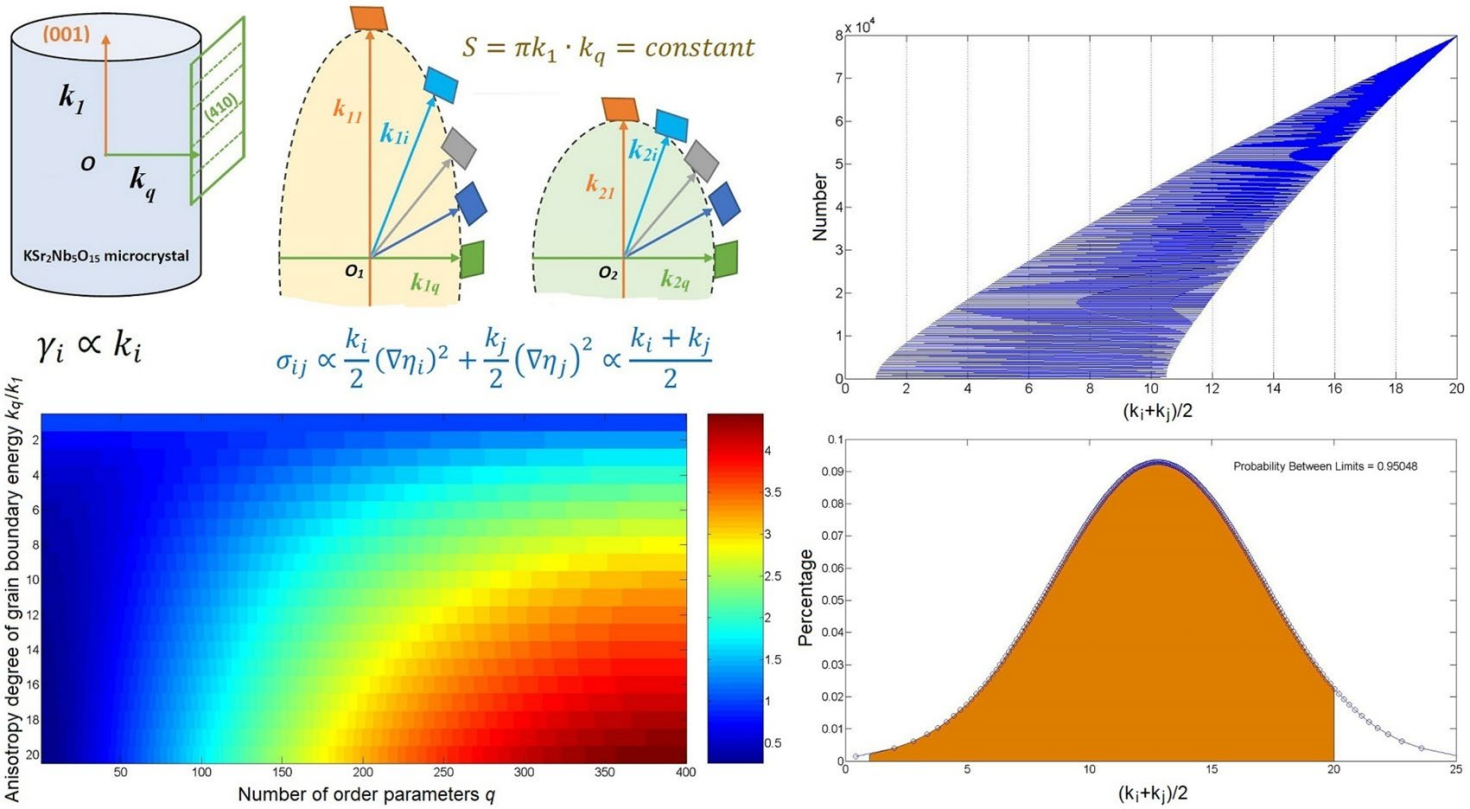
Schematic diagram illustrating the construction of anisotropy of the grain boundary energy.

The gradient energy coefficient *k*_*i*_ is proportional to the crystallographic plane energy $\gamma$. Figure 2 shows the construction process of grain boundary energy anisotropy. As a comparative experimental material, KSr_2_Nb_5_O_15_ (KSN) crystal has the highest crystallographic plane energy of (001) and lowest crystallographic plane energy of (410). The energies of the other crystallographic planes are between them according to the Gibbs Wulff theorem^41^. We suggest that the energy values of all the surfaces satisfy an elliptic distribution as observed from the morphology of KSr_2_Nb_5_O_15_ particle^9^. For comparison purposes, we set the area of the ellipse to be constant^35^:

3 $$S=\pi {k}_{1}{k}_{q}$$

The ratio of *k*_*q*_ to *k*_*1*_ reflects the degree of anisotropy, *i.e*. anisotropic coefficient *A*_c_. As seen from Fig. 2, when *q* = 400, *k*_1_ = 1 and *k*_400_ = 20, 80,000 kinds of grain boundaries are combined in the system, and the number of grain boundaries with different energies is normally distributed. It can be seen that the setting of the grain boundary energy is sufficient to describe the real situation. Thus, the modified phase-field model^35^ is very suitable for simulating the evolution of grain orientation and shape in ceramic thick film.

### Initial conditions

**Figure 3:**
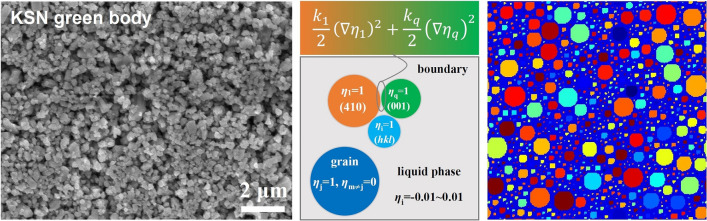
Generating process of the initial microstructure.

Figure 3 shows the SEM image of KSN thick film. As seen in the figure, the particles are arranged closely and the size distribution is uniform. We assume that the initial particles are circles with the same size distribution and random orientation distribution. The disjointed circles with the required size are randomly generated to fill the entire 256 × 256 grid-point system as shown in Fig. 3. Then, the LRO parameter values inside the circles are assigned as *η*_i_ = 1 and *η*_j≠i_ = 0, where *i* is a randomly natural number between 1 and *q*. Other regions are considered to be liquid-phase, *i.e. η*_i_ = − 0.01 ~ 0.01. Periodic boundary conditions are applied. The forward-Euler discretization scheme is adopted to discretize the time derivative in Eq. (1) ^39^:

4 $$\frac{d{\eta }_{i}}{dt}=\frac{{\eta }_{i}\left(t+\Delta t\right)-{\eta }_{i}\left(t\right)}{\Delta \text{t}}$$

The gradient term in Eq. (2) is evaluated as ^39^:

5 $${k}_{i}{\nabla }^{2}{\eta }_{i}={k}_{i}\sum_{j=1}^{8}\frac{{\eta }_{i}\left(\overrightarrow{{\mathbf{r}}_{j}}\right)-{\eta }_{i}\left(\overrightarrow{\mathbf{r}}\right)}{{\left(\Delta x\right)}^{2}}$$

where the $\overrightarrow{{\mathbf{r}}_{j}}$ terms represent the nearest neighbor positions of $\overrightarrow{\mathbf{r}}$. A set of *k*_*i*_ values for the expression of grain boundary energy anisotropy can be easily calculated by the Eq. (3). As can be seen from Eq. (5), the modified model in this work do not increase any computational workload. The value of *A*_c_ is set from 1 to 12. *L*_*i*_ = 1.0, *S* = 2π, $\Delta x = 2$, $\Delta t = 0.1$ 5. The time scale is represented by the time step (TS). TS = 1 corresponds to one $\Delta t$. To show the microstructural evolution, we define the function $Q(\mathbf{r})$ as

6 $$Q\left(\mathbf{r}\right)=\left\{\begin{array}{c}0.7 \sum_{i=1}^{q}{\eta }_{i}^{2}\left(\mathbf{r}\right)<0.7 \,  grain \,   boundary \\ \frac{q-i-1}{q}\sum_{i=1}^{q}{\eta }_{i}^{2}(\mathbf{r}) \,  inside \,  the \,  grain\end{array}\right.$$

The values of $Q(\mathbf{r})$ are shown in the color images. Different colors represent different grain orientations.

### Validation experiment

Three kinds of microcrystals were used as raw materials to prepare the ferroelectric ceramic thick films. The microcrystals were fabricated by molten salt synthesis (MSS) in the SrCO_3_–Nb_2_O_5_–KCl–NaCl system. The molar ratios of NaCl and KCl were 0, 0.5 and 1 (the corresponding ceramics were labeled as KSN, KNSN, and NSN). The mole ratio of SrCO_3_ to Nb_2_O_5_ was 0.8. The weight ratio of salt to oxides (SrCO_3_–Nb_2_O_5_) was 2. Details of the preparation of microcrystals have been reported previously^9^. The green films with a thickness of 30 μm were fabricated by tape casting^9,29^. To eliminate the influence of needle-like morphology, the microcrystals were first ball-milled for 24 h. Then, the solvent, binder, and 1 mol% Bi_2_O_3_ were added to the microcrystals powder and ball milling was continued for another 12 h. The binder and plasticizer were removed by heating the samples at 0.4 °C/min to 600 °C for 4 h. All green bodies were pre-sintered at 1180 °C prior to sintering at 1280 °C for 2 h in air. Samples for electron backscatter diffraction (EBSD) and scanning electron microscopy (SEM) (Tescan VEGA3 and Quanta 600 FEG) observation were prepared by cutting, machining and polishing with 0.25 μm diamond paste, followed by thermal etching for 30 min. The Phases of the samples were examined by XRD (Panalytical X′Pert PRO, Holland) using Cu Kα radiation and a graphite monochromator.

## Results and discussion

Generally, the energy of a grain boundary between crystal grains will be a function of the relative orientation of the two grains and the orientation of the boundary surface itself with respect to the two grains. For the anisotropic system, this means that two new parameters (misorientation *θ* and inclination angles *φ*) need be induced in the phase-field model. The addition of *θ* and *φ* makes the model more complex and introduces new requirements for computer memory and calculation methods. In the current study, we took advantage of a simple approach to describe the anisotropy of the grain-boundary energy. We can imagine building the grain boundary by joining the surfaces of the initial two particles. When the two surfaces are combined, new bonds are formed. Therefore, the grain boundary energy can be expressed as^42^

7 $${\sigma }_{gb}=\left({\gamma }_{s1}+{\gamma }_{s2}\right)-B$$

where *γ*_s_ is the surface energy of the initial particle and *B* is the binding energy. In the 2D system, the grain boundaries had no thickness. The grain boundary can then be considered a transition zone from one crystallographic plane to another. Thus, the grain-boundary energy should be the gradient energy of the crystallographic planes.

**Figure 4:**
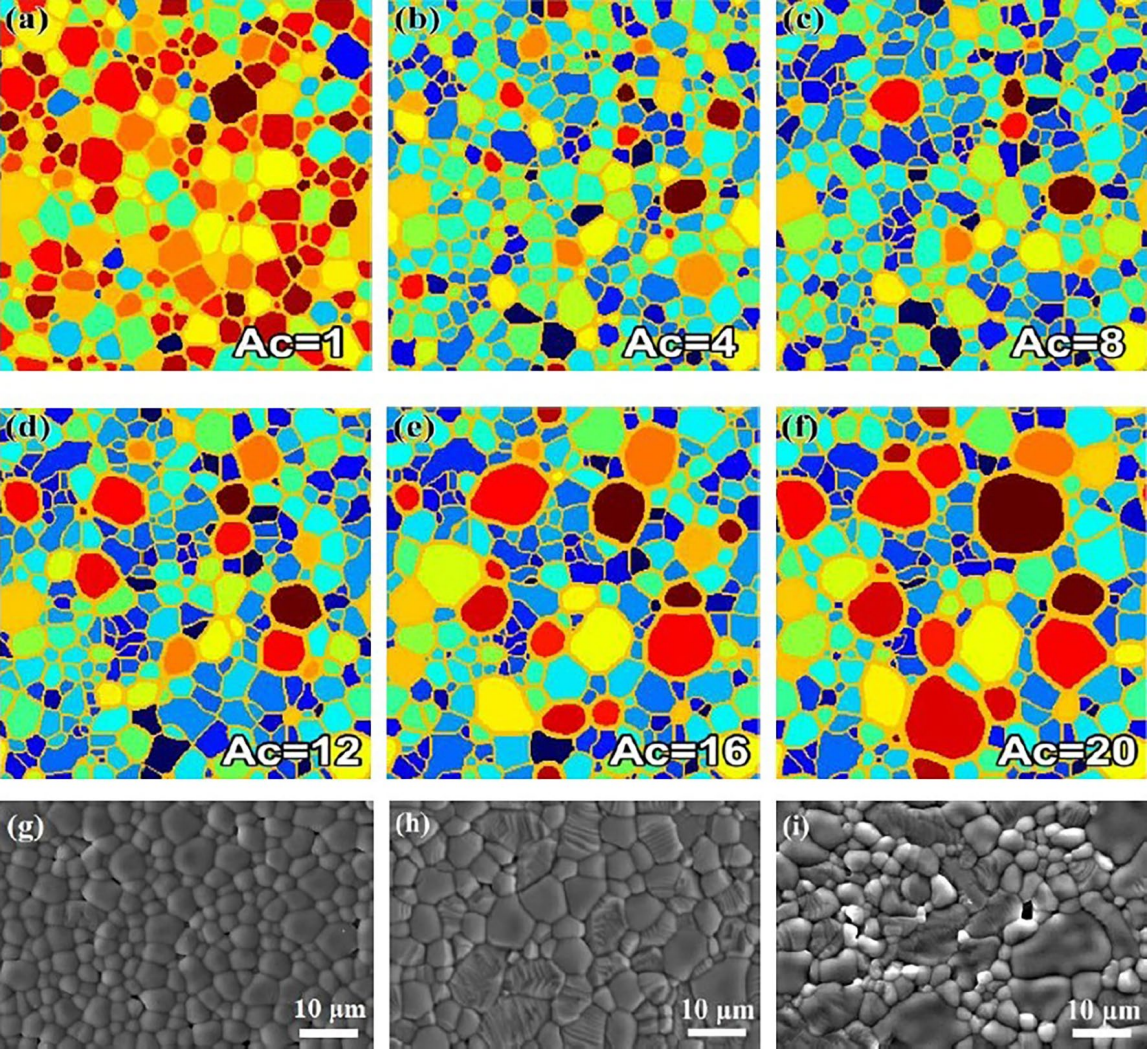
Microstructure evolution at TS = 2500 under the various conditions of grain boundary energy (**a**) *A*_*c*_ = 1, (**b**) *A*_*c*_ = 4, (**c**) *A*_*c*_ = 8, (**d**) *A*_*c*_ = 12, (**e**) *A*_*c*_ = 16, (**f**) *A*_*c*_ = 20, and SEM images of ferroelectric ceramic thick films (**g**) NSN, (**h**) KNSN, (**i**) KSN.

Using the above-mentioned computational conditions, we performed 2D simulations for the microstructure evolution. Figure 4 shows the grain microstructures at TS = 2500 under conditions of various grain boundary energies. As a comparative experiment, the case of isotropic grain boundary energy was also carried out. As shown in Fig. 4a, the grain size is uniform and the morphology of all grains is equiaxed. Colorful grains indicate that the orientation of grains is also evenly distributed. The simulation results are in complete agreement with the experimental results as reported in the literatures^4,7,8,10,11,13,18–20^. It can be concluded that the SEM image of functional ceramics with high crystal structure symmetry can be summarized as this microstructure^43,44^. This means that the energy of grain boundaries form by different oriented-grains is almost the same in these ceramics. However, for the cases of anisotropic grain boundary energy, the grain microstructures have changed significantly. When *A*_*c*_ = 4 (Fig. 4b), the number of blue grains increases and grain size decreases as compared with Fig. 4a. With the increase of *A*_*c*_, the red grains are not only coarsening in size, but also increasing in number. As the *A*_*c*_ value increases to 16 (Fig. 4e,f), three types of grain appear in the microstructures: (i) equiaxed grains, (ii) strip grains, and (iii) abnormally large grains. These phenomena indicate that the grain growth process is different under the conditions of anisotropic grain boundary energy, and closely related to the grain orientation.

Based on the growth mechanism of microcrystal in molten salt^45^, we concluded that the crystallographic plane energy of KSN crystal was strong anisotropy. With the substitution of K^+^ by Na^+^ in the A sites, the aspect ratio of the microcrystals decreased significantly^46^, which meant that the grain boundary energy anisotropy of NSN ceramics was relatively weak. For comparison with the simulation results, three kinds of the microcrystals were used to prepare thick films. As seen in Fig. 4g–i, the grain shape and size distribution of thick films ceramics are almost the same as that of bulk ceramics of their respective systems^9,16,17,32^. With the increase of the anisotropy degree of the crystallographic plane energy of raw materials, the grain size changes from unimodal to bimodal distribution. By comparing the SEM images with the simulation results, it can be seen that Fig. 4g–i are consistent with Fig. 4b, d and f, respectively. To verify the grain orientation distribution, we carried out the EBSD and XRD analysis of KNSN and KSN ceramics thick films. As observed from the EBSD images, the color of grains with different morphologies is also distinct. It can be seen that the equiaxed grains are close to blue, most of the strip grains are red, and the abnormally large grains are yellow-green. Although the errors of grain boundary in the EBSD images are large and not as regular as those in the SEM images, the grain orientation distribution is still reliable. In addition, the XRD patterns confirm that (001) area increased in the thick films. These results are also agree with the simulation results. So, the function of grain boundary energy constructed in this work is reasonable. In addition, under the condition of grain boundary energy anisotropy, grain orientation is an important parameter for microstructure evolution and must be considered in the study of grain growth dynamics.

**Figure 5:**
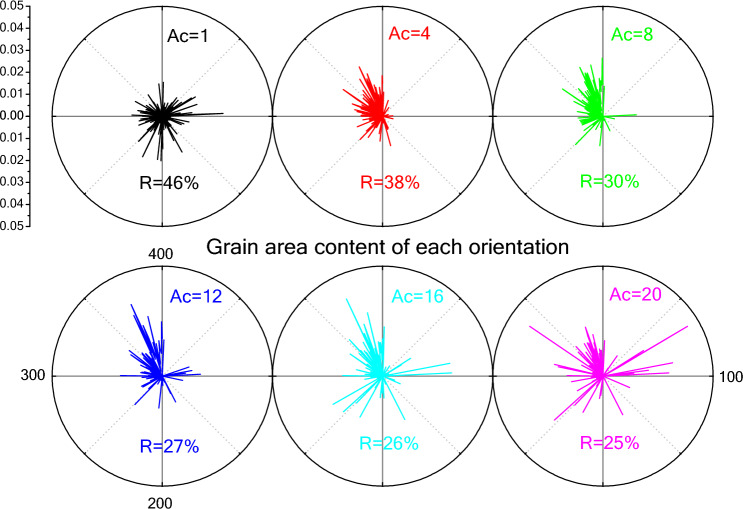
Grain orientation distributions at TS = 2500 under the various conditions of grain boundary energy (*A*_*c*_ = 1 ~ 20).

**Figure 6:**
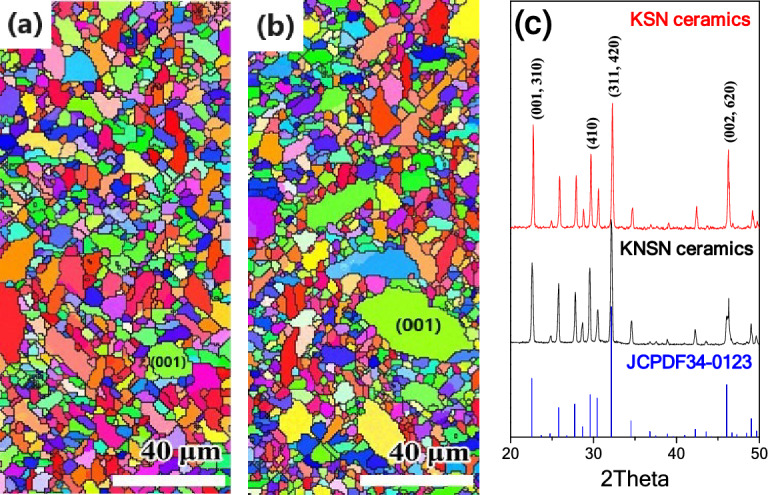
EBSD images (**a**, **b**) and XRD patterns (**c**) of the KNSN and KSN ceramics thick films.

To analyze the grain orientation distribution more directly, the total grain area corresponding to individual orientation is calculated. Figure 5 shows the grain orientation distributions at TS = 2500 under the various conditions of anisotropic grain boundary energy by means of polar graph. It can be seen that the lines are evenly distributed around the center of the circle when grain boundary energy is isotropic (*A*_*c*_ = 1). When *A*_*c*_ > 1, the grains with orientation number from 1 to 100 almost disappear. However, after the *A*_*c*_ value increased to 12, a small number of lines appear again in the first quadrant (the number of LRO ranged from 1 to 100), i.e. the regions with high crystallographic plane energy as described in Fig. 2. The grain area of individual orientation in the first and fourth quadrants increases rapidly for the case of *A*_*c*_ > 16. This suggests that the grain is preferentially oriented to grow. The number of grains in the intermediate plane energy state is small, which can also be seen from Figs. 4 and 6. To quantify the orientation degree, we calculate the ratio *R* of orientation number between TS = 0 and TS = 2500. It can be obtained that the number of grain orientations gradually decreases as the degree of anisotropy increases. When the anisotropy of grain boundary energy is very strong, the number of grain orientation is only about half of that in the case of isotropy.

**Figure 7:**
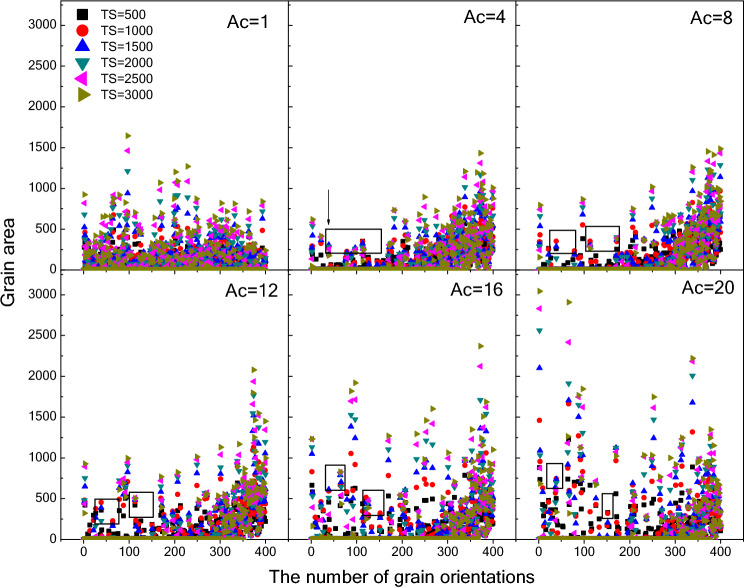
Changes in the grain area with the different orientations as a function of the time step.

Figure 7 shows the changes in the grain area with the different orientations as a function of the time step. It is noted that the orientation distribution of grains is independent of time step for the isotropic case (*A*_*c*_ = 1). This is because the energy of all grain boundaries is the same, and the growth or disappearance of grains has nothing to do with their orientation^40,41^. For the case of *A*_*c*_ = 4, a special area appears as marked by a rectangle. At the beginning of microstructure evolution, some grains with the same orientation grow gradually, but with the increase of time step, these grains shrink rapidly and disappear. The area become the diffuse distribution and individual grains are rapidly coarsened into the largest grains as the increase of *A*_*c*_. This phenomenon suggest that an increase in the degree of anisotropy of grain boundary energy is conducive to grain growth. In other words, the size and number of large grains in the thick films can be regulated by the anisotropy of crystal plane energy.

**Figure 8:**
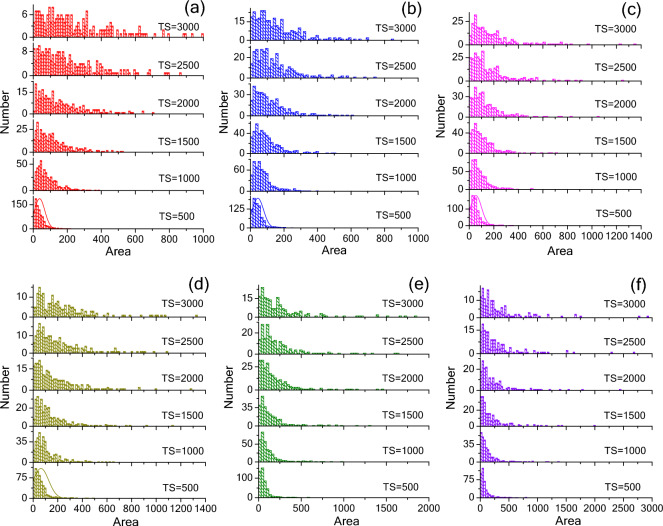
Grain size distribution under the various conditions of anisotropic grain boundary energy (**a**) *A*_*c*_ = 1, (**b**) *A*_*c*_ = 4, (**c**) *A*_*c*_ = 8, (**d**) *A*_*c*_ = 12, (**e**) *A*_*c*_ = 16, and (**f**) *A*_*c*_ = 20.

**Figure 9:**
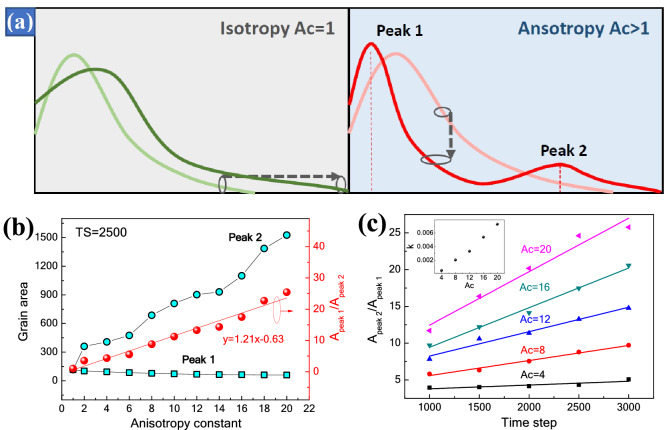
Schematic diagram of grain size distribution (**a**), changes in the area ratio of peak 1 and peak 2 as a function of the anisotropy constant (**b**) and time step (**c**).

Figure 8 shows the grain size distribution under the various conditions of anisotropic grain boundary energy. For the isotropic case (Fig. 8a), the initial curve of TS = 500 is normally distributed, and the peak is broadened and shifted along the large size direction as the time step increases. For the anisotropic cases (Fig. 8b–f), the curves gradually transfer from single peak to double peaks, and the position of the peaks is directly related to the *A*_*c*_ value. With the increase of *A*_*c*_, the small-size peak shifts to the small-size direction, and the large-size peak shifts to the large-size direction. This behavior results in the formation of a duplex structure as shown in Fig. 4. The duplex structure, *i.e*. no grain with an intermediate size, was often observed in ferroelectric ceramics^25,32^. Generally, the formation of this structure could be attributed to the inhomogeneous initial particle size^9^. The simulation results suggest that the anisotropy of crystal plane energy also plays an important role on the formation of a duplex structure. The change of grain size distribution with time step can be described as the change of a rigid rope under the action of tractive force as shown in Fig. 9a. As the time step increases, the rope extend in a horizontal direction. A change in the degree of anisotropy results in an increase in longitudinal tractive force. Thus, one peak is squeezed into two peaks, and the two peaks are moving further and further apart. Figure 9b and c show the changes in the area ratio of peak 1 and peak 2 as a function of the anisotropy constant and time step. It is interesting noted that the area ratio of peak 2 and peak 1 is directly proportional to the anisotropy constant, the relationship can be descripted as

8 $${A}_{peak 2}/{A}_{peak 1}=1.21{A}_{C}-0.63.$$

The ratios of ${A}_{peak 2}/{A}_{peak 1}$ are calculated by the statistics of grain size in Fig. 4g–i. The ratios are 5.35, 14.37 and 21.76, respectively. This indicates that the anisotropy constants for NSN, KNSN and KSN ceramics thick films are 3.9, 12.4 and 18.5. The ratio of ${A}_{peak 2}/{A}_{peak 1}$ also has a linear relationship with the TS, and the slope *k* is proportional to *A*_*c*_. Thus, we deduce the formula as follows

9 $${A}_{peak 2}/{A}_{peak 1}\propto {A}_{C}\cdot \text{TS}$$

Obviously, this relationship is suitable for the stage of continuous grain growth.

**Figure 10:**
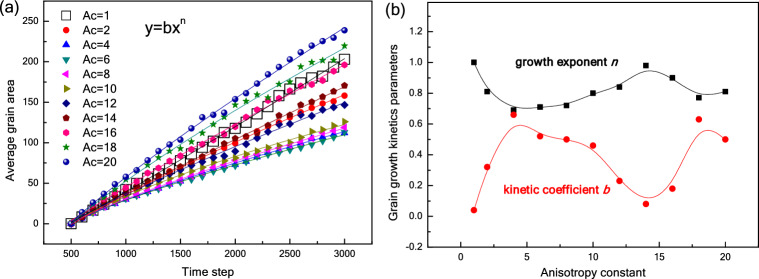
Kinetic curves of grain growth (**a**), and the changes in the growth exponent and kinetic coefficient as a function of anisotropy constant (**b**).

Figure 10 shows the kinetic curves of grain growth under the various conditions of anisotropic grain boundary energy. Fitted curves for the plots are calculated and depicted in Fig. 10a using the well-known parabolic law of steady-state grain growth^47^:

10 $${A}_{t}-{A}_{0}=b{t}^{n}$$

where *A*_0_ is the average grain area at TS = 500, *b* is the kinetic coefficient, and *n* is the growth exponent. For the isotropic case, *n* = 1. For the anisotropic cases, Fig. 10a reveals that the average grain area increases with time step in accordance with the parabolic law. It can be seen that the growth rate decreases and then increases (*A*_*c*_ > 6) with the increase of *A*_*c*_. Only when *A*_*c*_ = 16, the growth rate exceed that of the isotropic case (*A*_*c*_ = 1). This means that the weak anisotropy of grain boundary energy can inhibit grain growth owing to the formation of a large number of low energy grain boundaries (seen in Fig. 4). This may be the reason that some fine-grained microstructures can be obtained in the ferroelectric ceramics^19,20,48^. It can be seen in Fig. 10b that the change of growth exponent *n* presents W-shaped curve, and all *n* values are less than 1. The kinetic coefficient *b* shows the opposite variation pattern as *n*. According to the above analysis of Figs. 4, 7 and 8, it can be seen that the growth of grains with high crystal plane energy should be responsible for the W-shaped changes.

**Figure 11:**
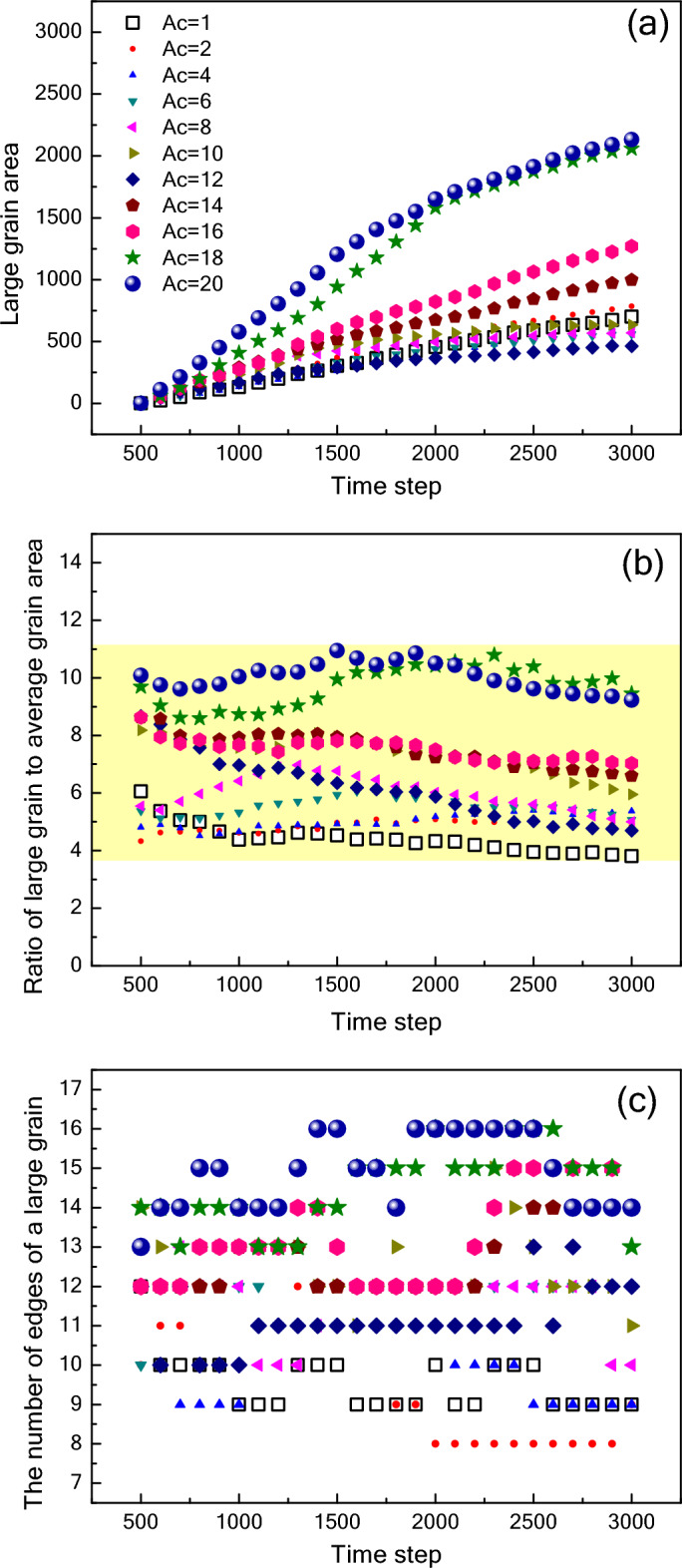
Changes in the large grain area (**a**), the ratio of a large grain to average grain area (**b**), and the number of edges of a large grain (**c**) with the different orientations as a function of the time step.

To investigate the growth behavior of a large grain, we first find the largest grain in the image of TS = 3000 as the target grain, and then track its growth process. It is found that the orientation of the large grains is similar (seen in Fig. 4), and all of them have high crystal plane energy. Figure 11 shows the size and shape change of large grain under the various conditions of anisotropic grain boundary energy. The growth rate of a given grain as a function of its size is proposed by Hillert^47^:

11 $$R\frac{dR}{dt}=0.5M\sigma \left(\frac{R}{\langle R\rangle }-1\right)$$

where < *R* > denotes the average grain radius, *M* is grain boundary mobility, and $\sigma$ is grain boundary energy. For the isotropic case ($\sigma$ of each grain was the same), the driving force is determined only by the radius of curvature of the grain boundary. Thus, the initial grains with different orientations have the same probability of becoming large grains. The large grain keeps growing steadily as shown in Fig. 11a. However, for the anisotropic cases, the driving force of grain growth is mainly controlled by grain boundary energy. Therefore, the grains with high crystal plane energy are likely to become the largest grain. As shown in Fig. 11a, the growth rate of grain decreases gradually with the increase of time step. Similar to the change trend of average grain area (Fig. 10a), the growth rate of grain decreases first and then increases with the increase of *A*_*c*_. As can be seen in Fig. 11b, the ratio of large grain to average grain area is between 4 and 11, and has a small range of fluctuations with the increase of time step.

It is well known that the growth rate of a given grain as a function of its topology (number of edges, *n*_e_) can be described by the von Neumann–Mullins law^49^:

12 $$R\frac{dR}{dt}=\frac{1}{6}M\sigma \left({n}_{e}-6\right)$$

We calculate the number of edges of a large grain at different time steps. It is found that *n*_e_ is independent of time step. In the isotropic case, the number of large grain edges is stable between 9 and 10. In the case of *A*_*c*_ = 2, the value of *n*_e_ drops to 8, although it reaches 12 at the beginning stage of grain growth. With the increase of *A*_*c*_, the value of *n*_e_ increases and reaches a maximum of 16 for the case of *A*_*c*_ = 16. For comparison, corresponding experimental results are calculated. Because the number of large grains is small as shown in Fig. 4g–i, we choose one grain with the largest number of surrounding grains and the number of surrounding grains as its edge number (*n*_e_). The values of *n*_e_ are 9, 11 and 14 for NSN, KNSN and KSN ceramics thick films, respectively. According to the results in Fig. 11c, we deduce that the corresponding *A*_*c*_ is considered to be in the range of 1 to 4 for NSN, 8 to 12 for KNSN, 14 to 20 for KSN. It is in complete agreement with the analysis results in Fig. 9.

**Figure 12:**
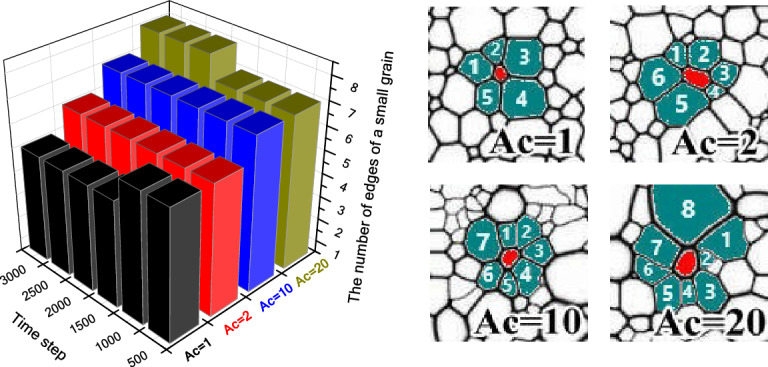
The number of edges of a small grain under the various conditions of grain boundary energy.

According to the analysis in Fig. 4, after the grain boundary energy changed from isotropic to anisotropic cases, the number of small grain edges (less than the average grain size) also changes significantly. It can be seen from Eq. (12) that when grain boundary energy is isotropic, the maximum number of small grain edges is 5. This phenomenon has been confirmed by experiment and simulation results^4,7–9,18–20,39,40^. Figure 12 shows the number of edges of a small grain under the various conditions of grain boundary energy. It is noted that as the value of *A*_*c*_ increases from 1 to 20, the number of edges of small grains increases from 5 to 8. In addition, the small grains with more edges (> 5) can be observed easily in the simulation images of *A*_*c*_ > 1. That is, with the increase of *A*_*c*_ value, the number of polygonal small grains (*n*_*e*_ > 5) increases. We have observed a large number of published SEM images of ferroelectric ceramics, and found that the maximum edge number of small grains was no more than 8.

The above results allow us to conclude that the anisotropy of grain boundary energy has a significant effect on grain size, distribution and orientation. Thus, the anisotropy degree of grain boundary energy can be judged by calculated the degree of orientation, the area ratio of peak 2 and peak 1, the ratio of the largest grain to average grain area, and the number of edges of large and small grains. All these information can be obtained from SEM or EBSD images.

## Conclusions

The phase-field model is employed to simulate the grain microstructure evolution of ferroelectric ceramic thick films. The anisotropy of grain boundary energy is constructed by the adjacent grains with different crystal plane energies. The effects of anisotropy of grain boundary energy on the grain orientation and shape evolution are investigated. It is found that the probability of becoming a large grain is closely related to its orientation. The number of grains with intermediate boundary energy is small. The grain size distribution is bimodal, and the area ratio of peak 2 and peak 1 increases with the increase of the degree of anisotropy of grain boundary energy. Three kinds of grains can be observed: (i) equiaxed grains, (ii) strip grains, and (iii) abnormally large grains. The number of edges of large grains is between 8 and 16. The maximum number of edges of small grains is 8. These results is consistent with the experimental observations, which will be helpful to obtain information about the anisotropy of grain boundary energy by analyzing the microstructure of ferroelectric ceramics. This work also confirms that the orientation of initial grain has an important effect on the microstructure of ferroelectric ceramics, also explains the reason why ferroelectric ceramics have different microstructures in 2D planes.

## Data Availability

The datasets used and/or analysed during the current study available from the corresponding author on reasonable request.
